# Enhancing Cleavage Aesthetics in Patients Seeking Breast Augmentation: Surgical Technique and Key Considerations

**DOI:** 10.1055/s-0045-1812105

**Published:** 2026-01-05

**Authors:** Rajat Gupta, Tanvi Rao, Priya Bansal, Gautam Chaudhury

**Affiliations:** 1 Department of Plastic Surgery, Excel Hospital, C.K. Birla Hospital, and Rosewalk Hospital, New Delhi, India; 2 Department of Plastic Surgery, Kasturba Medical College, Manipal, Manipal Academy of Higher Education, Manipal, Karnataka, India

**Keywords:** hybrid augmentation, breast, implants, fat grafting, breast aesthetics, cleavage, enhancement

## Abstract

**Background:**

Hybrid breast augmentation, a combination of silicone implants and autologous fat grafting, is an innovative approach aimed at achieving natural aesthetics. This study evaluates its impact on reducing intermammary distance and improving cleavage aesthetics.

**Materials and Methods:**

This article and the accompanying videos provide a detailed, step-by-step guide to hybrid breast augmentation using a subfascial inframammary approach, with a predictable and reproducible surgical outcome.

**Results:**

Postoperative cleavage distance decreased significantly (mean reduction from 9.5 ± 1.23 to 4.74 ± 0.48 cm, *p* < 0.0001). High patient satisfaction was noted, with 93.9% rating their experience as “very satisfied.” Minor complications (e.g., wide scars) were noted in only 3.1% of cases.

**Conclusion:**

Hybrid breast augmentation effectively reduces intermammary distance, enhances cleavage, and achieves high patient satisfaction with minimal complications. This method represents a reliable technique for improving breast aesthetics, warranting further exploration in diverse patient populations.

## Introduction

Breast augmentation is one of the most commonly performed cosmetic procedures worldwide, with continual advancements in techniques, materials, and outcomes. It is primarily undertaken to enhance breast volume, correct asymmetry, or restore breast shape following pregnancy or weight loss. 1 Innovations in surgical approaches have led to improved aesthetic results, shorter recovery times, and decreased complication rates. 2

Achieving optimal outcomes in breast augmentation relies on comprehensive preoperative planning, precise surgical execution, and attentive postoperative care—all within a structured, stepwise framework to maximize patient satisfaction. 3 Despite ongoing discussions regarding implant types, incision techniques, and long-term safety, preferences among patients and surgeons are widely influenced by cultural and demographic factors. 4 5

Traditional implant-based augmentation has certain limitations, such as the inability to modify the breast footprint or intermammary distance, and the potential for an unnatural appearance when there is an imbalance between soft tissue coverage and implant size. 6 7 8

Hybrid breast augmentation (HBA), which combines implants with autologous fat grafting (AFG), has emerged as a valuable technique that addresses these issues by enhancing volume while also refining contour and cleavage definition. 9 10 By supplementing soft tissue coverage around the implant, HBA achieves a more natural look. Furthermore, because smaller, well-distributed volumes of fat are used, the incidence of fat grafting–related complications appears to be reduced compared to AFG alone. 11 Careful patient selection and standardized fat grafting zones are essential to ensure safety and effectiveness. 12

HBA transitions the traditionally steep implant contour into a smoother, more natural slope, improving the overall breast shape and enabling better cleavage formation. This article presents a step-by-step surgical video that demonstrates technical details, including incision entry points, planes of fat placement, and recommended timing and volume of fat transfer. It aims to enhance surgical consistency and deepen understanding among practitioners seeking to adopt or refine this technique.

## Materials and Methods

Between January 2021 and September 2024, 131 female patients presented with concerns about aesthetically unsatisfactory breasts and underwent HBA. A retrospective study was conducted on this cohort. All procedures were performed at a single center by the same surgical team to ensure consistency. The study was approved by the GeneBandhu ethics committee (Ref- ECG005/2025). The meeting was held on January 17, 2025.

## Surgical Technique

### Patient Selection and Preoperative Markings

**Video 1** Pre-operative markings for hybrid breast augmentation.


Preoperative markings were completed with the patient in the standing position, as shown in Video 1 . Key anatomical landmarks, including the sternal notch and midline, were identified and marked first. Symmetrical breast meridians were drawn on both sides, and the inframammary creases (IMCs) were delineated bilaterally.

From the medial edge of the nipple, a reference point was marked on the IMC. A planned 3 cm incision was then marked, positioned two-thirds laterally and one-third medially from this reference point on the inframammary fold. The incision was carefully placed to ensure maximum concealment for better aesthetic outcomes.

Using a vernier caliper, the implant's base width plus 1 cm was measured. This measurement guided the marking of the dissection limits on the right breast, including the superior, inferior, medial, and lateral boundaries.

On the opposite breast, the superior and inferior limits were similarly marked. To ensure symmetry, the distance between the medial limit of the right breast and the midline was measured using a flexible scale. This measurement was mirrored on the left breast to mark its medial limit. From this medial marking, the lateral limit was determined using the caliper measurement.

Finally, the medial cleavage boundaries are marked for auto fat grafting.

This systematic marking process ensured accurate and symmetrical placement of all incisions and dissection boundaries. Upper pole fat grafting was performed selectively in only a few cases where it was indicated. In most cases, fat grafting was limited to the cleavage area to reduce the intermammary distance.

### Inframammary Approach and Subfascial Pocket Creation

**Video 2** Pre-operative markings for hybrid breast augmentation.


All procedures were performed under general anesthesia. As a prophylactic measure, a single dose of 1 g ceftriaxone was administered intravenously 1 hour before surgery.

The incision was made along the IMC in accordance with the preoperative markings. The incision was deepened, and dissection proceeded until the pectoralis major muscle was reached. A subfascial pocket was then carefully created in an upward direction using a long-tip monopolar cautery. This dissection continues until the upper pole of the breast is reached, as defined during preoperative planning.

The IMC fixation was done using polydioxanone sutures. These scarpofascial sutures were anchored from the fascia over the chest wall to the Scarpa's fascia above. This step was essential to prevent bottoming out and to provide adequate support for the implant to be placed as shown in Video 2 .

The pocket was thoroughly irrigated with a triple-antibiotic betadine solution, consisting of a third-generation cephalosporin, aminoglycoside, and bacitracin. Hemostasis was meticulously achieved, and the pocket was packed with a sterile mop. The same procedure was then repeated on the contralateral side.

After successfully creating the bilateral pockets, the surgery proceeded to the next stage.

### Implant Placement

The mops placed in the pockets were carefully removed, and hemostasis was confirmed. Preoperatively measured silicone implants were opened under sterile conditions and irrigated with a triple-antibiotic betadine solution. Using a funnel and adhering to the “No Touch” technique, the implant was gently positioned into the prepared pocket. Slight overcorrection was performed to compensate for anticipated fat resorption.

The same steps were meticulously repeated on the contralateral side. Finally, the incision was closed in layers to ensure optimal healing and structural integrity.

### Fat Harvest from the Abdomen or Thigh

**Video 3** Pre-operative markings for hybrid breast augmentation.


Depending on the patient's body habitus and patient preference, small to moderate amounts of fat tissue is harvested from the abdominal or thigh area following tumescent infiltration as shown in Video 3 . Harvesting was performed using a 2.4-mm cannula, and the fat was processed and prepared for grafting. Lignocaine was included in the infiltration fluid; however, the harvested fat was thoroughly washed multiple times to minimize any residual lignocaine and preserve graft viability.

### Fat Injection into the Breast

Fat grafting was performed in the medial and superomedial quadrant of the breast using a fan-like pattern to enhance cleavage and achieve a natural medial transition. A 10-mL Luer-Lock syringe connected to a 1.2-mm bulb-tip cannula was employed to ensure precise delivery. An 18G needle was used to make a pinpoint hole and fat was carefully injected into the pre-marked area in the subcutaneous plane until optimal coverage was achieved.

The volume of fat injected per breast ranged from 50 to 100 mL, enhancing the contour of the breast cleavage and reducing the intermammary distance for improved aesthetic outcomes.

### Postoperative Care

**Fig. 1:**
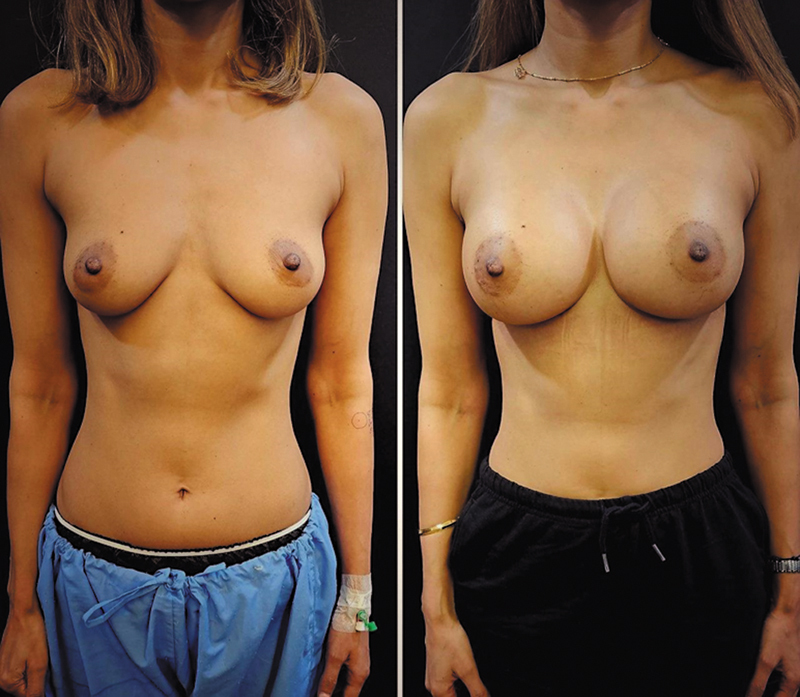
Before and after images of a 27-year-old, 6 months after 285 cc smooth high-profile implant in the subfascial plane, with 55 cc fat grafting on each side.

Patients were ambulated within 4 hours postoperatively and discharged with compression garments over the fat graft harvest areas and sports bras over the breasts ( Figs. 1 2 3 4 ). A sports bra was recommended to be used for 6 weeks. Heavy lifting was avoided for 4 weeks.

**Fig. 2:**
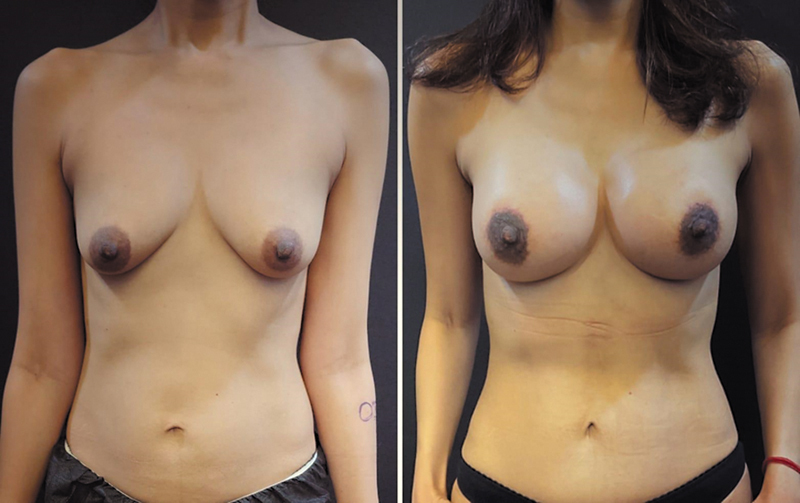
Before and after images of a 38-year-old tuberous breast deformity correction, 6 months after 275 cc smooth high-profile implant in the subfascial plane, with 75 cc fat grafting on each side.

**Fig. 3:**
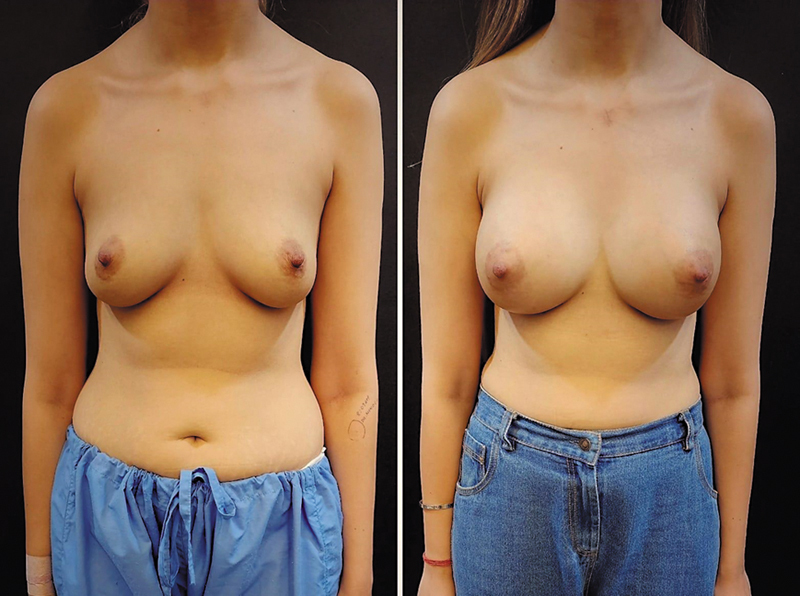
Before and after images of a 34-year-old, 6 months after 235 cc smooth high-profile implant in the subfascial plane, with 45 cc fat grafting on each side.

**Fig. 4:**
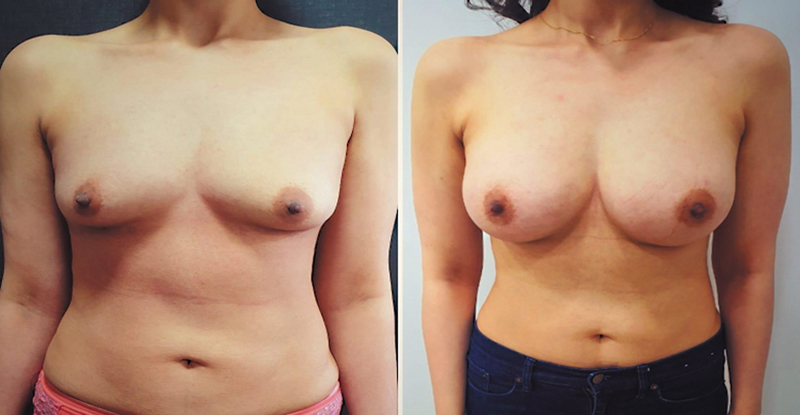
Before and after images of a 33-year-old, 6 months after 305 cc smooth high-profile implant in the subfascial plane, with 40 cc fat grafting on each side.

## Data Collection and Statistical Analysis

Data were collected on patient demographics (age), preoperative and postoperative BREAST-Q scores, intermammary distance measurements (baseline and 3 months postoperatively), and postoperative complications.

Follow-up evaluations were conducted at 48 hours, 1 week, 3 weeks, 3 months, and 6 months, with additional visits scheduled as needed for reported complications.

Statistical analysis was performed to compare preoperative and postoperative BREAST-Q scores. The mean scores for each domain of the questionnaire were calculated and compared using a paired *t* -test.

## Results

A total of 131 patients undergoing HBA were followed up for 6 months, yielding satisfactory results in terms of achieving enhanced shape and less visible scarring.

The age of the patients ranged from 30 to over 55 years, with a mean age of 46.5 ± 5.3 years. Most patients were in the 41 to 45 age group (32.1%) and 46 to 50 age group (31.3%), reflecting a preference for the procedure among individuals in midlife.

Cleavage distance was measured both before surgery and 3 months after the procedure. Postoperative outcomes were favorable, with a mean cleavage distance of 9.5 ± 1.23 cm preoperatively, ranging from 8 to 13 cm. The postoperative cleavage distance was reduced to 4.74 ± 0.48 cm, ranging from 3.4 to 5.8 cm, demonstrating consistent and aesthetically pleasing results ( Table 1 ). The change in cleavage distance was statistically significant ( *p* < 0.0001).

**Table 1:** Cleavage distance

Cleavage distance (cm)	Preoperative	Postoperative
**Mean ± SD**	9.5 ± 1.23	4.74 ± 0.48
**Range**	13–8 cm	5.8–3.4 cm
**Median (IQR)**	9 (1.5)	4.7 (0.6)

Abbreviations: IQR, interquartile range; SD, standard deviation.

Note: *p* -value < 0.0001.

The procedure demonstrated excellent safety, with no major complications reported in 96.9% of cases. A small percentage of patients (3.1%) developed scars wider than 2 mm. All of these patients had a prior history of hypertrophic scarring and were successfully managed with intralesional tricort. This was the only complication observed.

Patient satisfaction was high, with 84.0% of patients dissatisfied preoperatively (score 0–50) and 93.9% highly satisfied postoperatively (score 76–100; Table 2 ). The mean preoperative satisfaction score was 42.88 ± 19.3, while the mean postoperative score was 84.83 ± 7.6, statistically highly significant improvement ( *p* < 0.0001).

**Table 2:** Patient satisfaction based on BREAST-Q

Satisfaction level	Preoperative (frequency)	Percentage	Postoperative (frequency)	Percentage
**Dissatisfied (0–50)**	110	84.0	1	0.8
**Satisfied (51–75)**	21	16.0	7	5.3
**Highly** **satisfied (76–100)**	0	0	123	93.9
**Mean ± SD**	42.88 ± 19.3		84.83 ± 7.6	

Abbreviation: SD, standard deviation.

Note: *p* -value < 0.0001.

## Discussion

AFG has been extensively utilized in facial rejuvenation, with its use in breast surgery gradually gaining acceptance, particularly after the American Society of Plastic Surgeons lifted its moratorium in 2008. Historically introduced by Neuber and later reintroduced by Bircoll in 1987 for breast augmentation, fat grafting has emerged as a reliable soft tissue filler for both small- and large-volume applications. 13

HBA integrates implants for core volume and fat for contour refinement, offering personalization in shape, projection, and symmetry. This composite approach facilitates a natural aesthetic by softening implant transitions and enhancing cleavage and upper pole fullness. 14

Ideal for patients desiring natural-appearing breasts with adequate projection, HBA allows for correction of asymmetries and improved contour through differential fat grafting. Fat transfers soften the steep curve of implant take-off, providing a smoother, more natural transition on the breast mound. This technique is especially appreciated by patients desiring a natural silhouette without sacrificing the firmness and projection offered by implants.

HBA is an innovative approach in aesthetic surgery, combining the structural benefits of implants with the contouring advantages of AFG, which allows precise enhancement of breast contours and cleavage. The flexibility of fat grafting enables individualized approaches, ensuring tailored outcomes for patients with diverse anatomical features and aesthetic goals. 15

Our results align with results from similar studies, showing the effectiveness of hybrid augmentation in achieving natural-looking outcomes. 16 Implant sizes ranged from 230 to 320 cc, and fat grafting volumes varied between 50 and 100 cc, demonstrating the reliability of the technique in meeting individual patient needs. 17

The safety profile of the subfascial hybrid technique was good, with no major complications reported in 96.9% of cases. A small percentage (3.1%) experienced minor complications, such as wide scars, which were easily managed. The use of triple-antibiotic irrigation and meticulous hemostasis during surgery likely contributed to the low incidence of complications. These findings are consistent with other studies highlighting the safety and reliability of hybrid approaches. 11 12

Patient satisfaction was high, with 93.9% of patients reporting scores in the 76 to 100 range postoperatively, compared to only 16.0% preoperatively. This statistically significant improvement ( *p* < 0.0001) shows the success of the procedure in addressing patients' aesthetic concerns. The combination of subfascial implant placement and precise fat grafting has proven to be a powerful technique for achieving both structural and aesthetic goals. As this approach continues to evolve, further research and refinement will enhance its utility and expand its applications in both primary and revision breast augmentation settings. 9 10 Based on our observations, even when the fat was absorbed, it formed a tissue scaffold that elevated the skin slightly, creating a gentle slope in the medial pole and thereby enhancing overall cleavage aesthetics. 18

Patient-reported outcomes were assessed using the BREAST-Q questionnaire, a validated and widely adopted tool for breast surgery outcomes. 19

However, to truly understand its full potential, further studies with diverse implant types and a broader patient population are crucial.

This study has certain limitations. Being retrospective in nature, it is subject to inherent selection and documentation bias. The relatively small sample size and single-center design may restrict the generalizability of the findings. Furthermore, long-term durability of outcomes and late complications could not be evaluated. Future prospective, multicenter studies with larger cohorts and extended follow-up are required to validate these results.

## Conclusion

By combining implants with fat grafting, we achieve a balance between projection and a natural look. Fat grafting enhances key aesthetic features—softens the intermammary cleft, creates fullness in the upper and medial poles, and ensures perfect symmetry between the breasts.
